# Infection of dogs with *Echinococcus granulosus*: causes and consequences in an hyperendemic area

**DOI:** 10.1186/s13071-015-0832-3

**Published:** 2015-04-17

**Authors:** Raja Chaâbane-Banaoues, Myriam Oudni-M’rad, Jacques Cabaret, Selim M’rad, Habib Mezhoud, Hamouda Babba

**Affiliations:** LP3M: Laboratoire de Parasitologie-Mycologie Médicale et Moléculaire, LR12ES08, Faculté de Pharmacie, Université de Monastir, 1 rue Avicenne, Département de biologie clinique B, 5000 Monastir, Tunisie; INRA and Université F. Rabelais, UMR 1282, Infectiologie et santé publique, 37380 Nouzilly, France; Laboratoire de parasitologie, E.P.S F. Bourguiba, 5000 Monastir, Tunisie

**Keywords:** *Echinococcus granulosus*, Dog faecal sample, Genetic variability, Environmental contamination, G1 genotype, Tunisia

## Abstract

**Background:**

Tunisia is a hyper endemic country for human echinococcosis. The infection is transmitted via the eggs of *Echinococcus granulosus* which are passed in the faeces of the definitive canid host.

**Methods:**

This study evaluated the contamination rate of the dog faeces in different climatic conditions at eight different geographic regions throughout Tunisia. Dog faecal samples were collected from the soil and the *Echinococcus* eggs were identified using microscopic and molecular (Eg1121/1122 PCR, Egss1 PCR and Nad1 PCR-RFLP) tools.

**Results:**

The contamination index of dog faeces by *E. granulosus* eggs ranged from 8.3% to 41.3% depending on the region. Comparisons of the dog faecal contamination rate against human incidence found them to be independent. Neither human prevalence nor dog contamination index appeared to be related to climatic conditions or geographic characteristics. The genetic variability of *E. granulosus* samples was different within each region but was not related to geographic distance which is indicative of local divergent evolutions rather than isolation by distance.

**Conclusions:**

A high environmental dog contamination index does not necessarily correspond to high prevalence in humans as transmission is strongly linked to human behavior and hygiene.

## Background

Echinococcosis is a widespread zoonotic parasitic disease which has major medical and socio-economic costs for humans and also threatens livestock productivity [1,2]. The causal pathogen, *Echinococcus granulosus*, parasitizes canids as its definitive hosts where the adult cestode inhabits the small intestine. Herbivores act as the intermediate hosts for the parasitic larval stage (metacestode) which is most commonly found in the host lungs and/or liver [3]. Human contamination occurs following the ingestion of taeniid eggs (or eggs reduced to embryophores) through contaminated food, essentially vegetables and water [4] or by direct contact with contaminated dogs that retain eggs on their coat [5]. This leads to the development of cystic echinococcosis (CE). Tunisia is considered as an echinococcosis endemic region with an annual surgical incidence (*SI*) of 12.6 cases per 100,000 inhabitants (*SI =* 12.6) [6] and approximately US$ 10–19 million losses annually in both humans and animals [7]. Other Mediterranean countries such as Algeria (*SI =* 3.6*-*4.6), Libya (*SI =* 4.2), Morocco (*SI =* 4.55), Italy (*SI =* 1.6), Spain (*SI =* 0.36), Greece (0.2) and France (*SI* =0.1) have a lower surgical incidence [8-10]. However, the endemic status in Tunisia differs from one region to another. Some areas have been defined as hyperendemic (*SI* >22.6) such as the West-northern regions of Kasserine and Kef, others as mesoendemic (7.5 < *SI* <15) such as the eastern region of Sousse and the region of Metlaoui situated in the south of Tunisia, and finally some as hypoendemic regions (*SI* < 7.5) for the east-central region of Monastir, the south eastern regions of Zarsis, Djerba and Tataouine [6]. *E. granulosus* is a complex in which four or five cryptic species are intermixed. Thus, *E. granulosus sensu lato* was split into *E. granulosus sensu stricto* (genotypes G1 to G3), *E. equinus* (genotype G4), *E. ortleppi* (genotype G5), *E. canadensis* (genotypes G6 to G10) and *Echinococcus felidis* (lion strain) [11-13]. Only four genotypes have been described in Tunisia: the G1 genotype in humans, sheep, cattle and dromedaries [14,15], the G6 genotype in the Southern dromedaries [16], the G3 genotype in one cattle and one human isolate [16] and the G4 genotype in donkeys [17]. CE was reported in livestock and the prevalence of infection was 16.42%, 8.56%, 5.94% and 2.88% in sheep, cattle, dromedaries and goats respectively [18]. In Tunisia, the canine population is composed essentially of stray and semi-stray dogs (free-roming dogs which are fed by an owner), and rarely receives deworming treatment [19]. In rural areas, 80% of households own at least one dog. The canine density is one per 3.0 to 5.5 inhabitants. There are 7 to 30 dogs per km^2^ according to the regions [20]. A high prevalence of *E. granulosus* infection has been reported in Tunisian dogs ranging from 19 to 45.7% in function of the regions [21,22]. Although the infection is essentially propagated by dogs in areas where they are kept at home, the persistence of the parasite life cycle is linked to the durability of the *E. granulosus* eggs in the environment in places where stray dogs are a majority. The eggs remain infective to humans and the intermediate hosts for a long time (nearly four years) after having been deposited onto the soil and under different climatic conditions [23]. Determining the prevalence at which eggs are shed into the environment and their capacity to survive is fundamental to ascertain the real endemic status of echinococcosis in an area [24,25]. In dogs, the prevalence is suggested to be high (up to 65%) in most Mediterranean countries [26]. However, these calculations were based on worms collected at necropsy from a limited number of dogs resulting in large confidence intervals. For example, a prevalence of 59% (46–71%) was detected in Morocco [27] and 7% (3-17%) in Tunisia-Sidi Bouzid [19]. Several techniques exist to assess the prevalence of *E. granulosus* in dogs including detection of worm antigen in faeces (coproantigen) [28], worms at necropsy [29] or direct examination of eggs in dog faeces [30]; however, these may vary in their estimates of prevalence. The direct sampling of dog faeces represents a relatively strong diagnostic tool as the taeniid eggs are easily recognized by light microscopy and molecular tools permit the specific identification of *E. granulosus* [31-33]. Although several epidemiological studies were performed on prevalence in necropsied dogs in Tunisia [21,22] no information on the actual contamination of the environment by *E. granulosus* eggs is available. Our objectives were to: 1) assess the contamination index of *E. granulosus* eggs in dog faeces in regions of differing endemicity in Tunisia in order to determine the level of environmental contamination; 2) explore factors which may explain differences in the contamination index of dogs between regions including: environmental influences on egg development, the density of intermediate hosts and the possibility of worm transfer between regions as based on the genetic variability, 3) and finally relate the human incidence with dog contamination index.

## Methods

### Sampling of dog faeces

**Figure 1:**
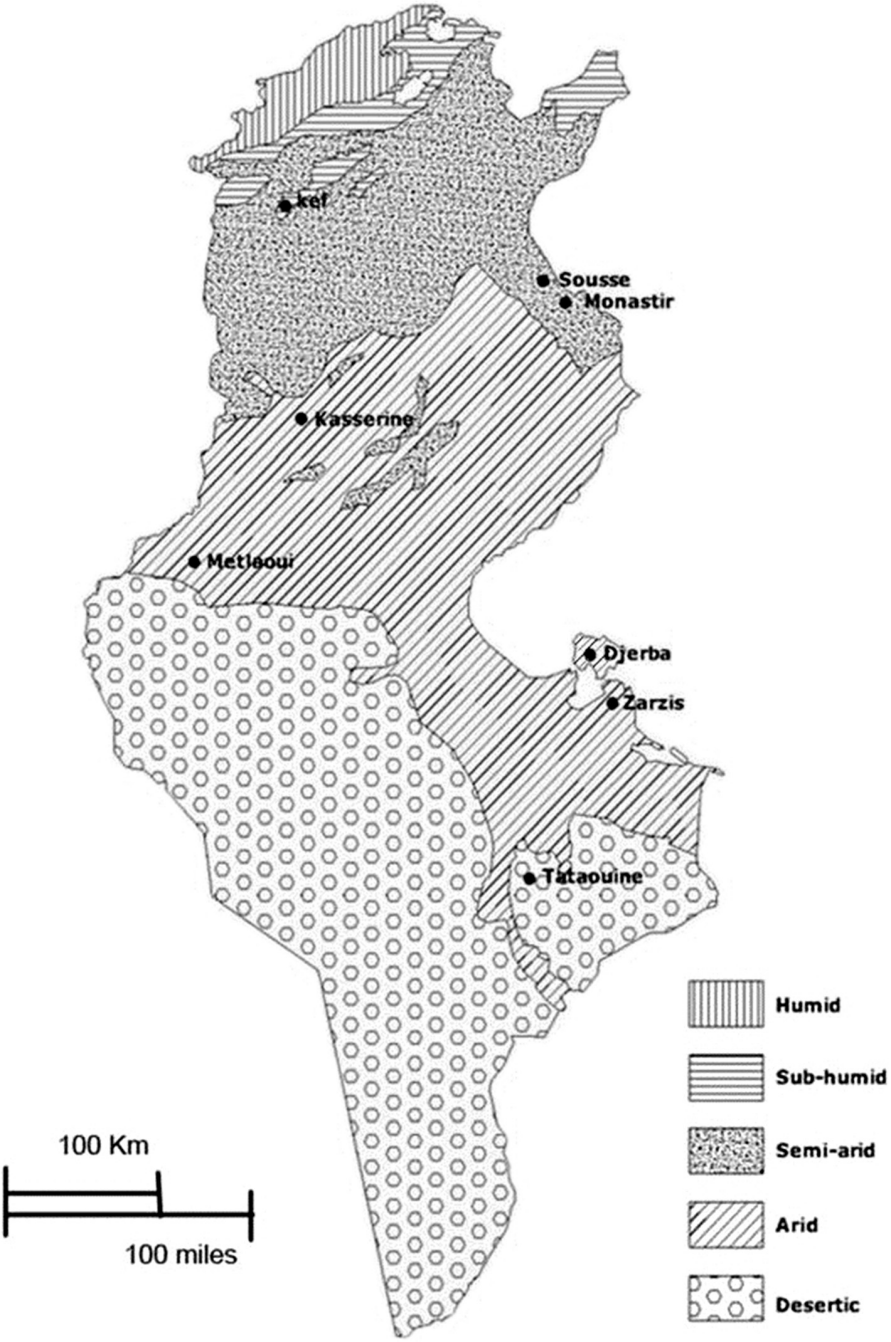
Dog faecal sample collections (eight sites) and their locations within the different climates of Tunisia.

One thousand ninety five dog faecal samples were collected from four different climatic zones in Tunisia (Figure 1): Kef (sub-humid), Monastir and Sousse (semi-arid), Metlaoui, Kasserine, Zarzis and Djerba (arid), and Tataouine (desert). These are all rural areas where livestock-farming occurs and the presence of stray and semi-feral dogs was observed. Forty samples were collected from a proportion of the faeces observed over a soil surface of 200-400 m^2^ on each location. The sampling was not related to a number of individual dogs but intended to represent the available eggs of *Echinococcus granulosus* on the area. Several sites were visited for each region (1–5) and collected during the Spring and Summer. The faecal samples were frozen at −80°C for 7 days in order to partially inactivate infective stages of the parasites [34] and then stored at −20°C until use. The parasite eggs were recovered from faecal samples using a flotation technique in modified Sheather’s solution (specific gravity: d = 1.27) followed by centrifugation at 1200 x g [35]. The taeniid eggs were subsequently identified morphologically [36]. The eggs were collected, out from the coverslip, using a 0.9% NaCl solution and stored at −20°C. Nevertheless, the taeniid eggs are morphologically indistinguishable and the *E. granulosus* egg identification requires the use of PCR technique [31]. The sampling was not affecting dogs or their owner since they were collected in the wild and thus no special notification to an ethical committee was required.

### DNA extraction

An alkaline lysis was performed to destroy the embryophore’s rigid shell [37]. Briefly, 14 μl of DTT (dithiothreitol) (1 mol/l) and 50 μl of KOH (potassium hydroxide) (1 mol/l) were added to 100 μl of egg suspension. The sample was heated at 65°C for one hour then neutralized with 120 μl of Tris–HCl (pH 8.3, 2 mol/l) and 10 μl of HCl (10 M). One hundred μl of lysis solution (Tris–HCl 50 mmol/l, pH 8, NaCl 100 mmol/l, EDTA 50 mmol/l, pH 8 and SDS 1%) and 10 μl of Proteinase K (Invitrogen) (20 mg/μl) were added to the sample and incubated for one hour at 65°C. Finally, the total DNA was extracted using a phenol-chloroform protocol [38].

### PCR amplification

The *E. granulosus* DNA was analysed by Eg1121/1122 PCR which amplified a 133 base pairs (bp) fragment of the tandem repeat EgG1*Hae*III [39] following the protocol of Naidich *et al.,* [32] modified as follows: the MgCl_2_ concentration was increased up to 3 mmol/l and the BSA (Bovine Serum Albumin) (0.1 mg/ml) was replaced by 1% formamide solution (Invitrogen). The percentage of dog faeces samples found positive for *E. granulosus* using PCR diagnostic provided the environmental contamination index by *E. granulosus*. The index of contamination of *E. granulosus* eggs was estimated as the number of PCR positive isolates/total number of examined samples in each region. Eg1121/1122 PCR is the most species-specific for *E. granulosus* but a cross reaction with the Tibetan wildlife species *E. shiquicus* was noted [40]. This should not be considered as a diagnostic problem since the presence of this species in Tunisia is highly improbable. The expected patterns of the amplification bands demonstrated the tandem arrangement of the EgG1*Hae*III repeat. The size of the major bands obtained matched the 133, 402, 671 and 940 bp and minor bands approximately at 300 and 600 bp. Amplified bands are larger by increments of 269 bp (the size of the repeat unit) [39]. Because the simultaneous existence of more than one genotype has been described in dogs [41], the PCR-RFLP method of the nad1 gene described by Hüttner *et al.* [42] was used to identify the genotypes of *E. granulosus* implied. A 1073–1078 bp-long fragment including the complete NADH dehydrogenase subunit 1 (nad1) gene was amplified and subsequently digested with the restriction enzyme HphI (New England BioLabs). The RFLP banding patterns allow a clear discrimination between *E. granulosus sensu stricto*, *E. equinus*, *E. ortleppi*, *E. canadensis* G6/G7 and *E. felidis* species. The presence of the genotype G1, which is the major genotype in Tunisia, was confirmed using Egss1 PCR [43]. This PCR amplified a mitochondrial sequence of 254 bp encoding the 12S rRNA small subunit.

### Statistical analyses

#### Characteristics of regions

The relationships between characteristics were established using non-parametric Spearman rank correlations. The contamination index of dog faeces and incidence in humans (the number of new cases per year), climatic characteristics and livestock density of studied regions were analysed using a principal component analysis-PCA with MVSP software (Multivariate statistical package. MVSP. User’ manual. Version 3.1. KCS, 288. Pentraeth, Wales, UK. 2002). The inertia values of each axis represented the percentage of variance, e.g. the explanatory power of the analysis. The end of vector (variable) location was indicative of its importance: at the intersection of the two axes it means that it has no explanatory power whereas when located far from the origin it means it is an important variable in the system. If variables were located in the same area of the graph, it indicates they were highly similar and positively related, if the variables were located in the opposite parts of the graph, they were negatively related. The annual surgical incidence [6] in humans and the dog faecal contamination index observed here were compared against climatic and geographic characteristics of studied regions [44,45] (Table 1). The contamination indices of faeces of the eight regions were analysed with Chi-square test (significance level at p < 0.05).

**Table 1:** **Bioclimatic and geographic characteristics of the studied regions**

**Region**	**Area (Km** ^**2**^ **)**	**Sheepd**	**Goatd**	**Cattled**	**Temp (C°)**	**Rain (mm)**	**Echhuman (ASI)**
Sousse	266200	0.68	0.02	0.05	18.3	300	9.88
Monastir	102400	1.17	0.03	0.14	18.3	300	6.22
Metlaoui	111335	0.16	0.00	0.00	19.3	106	11.04
Kef	42890	1.51	0.10	0.09	17.0	450	32.78
Djerba	53389	0.24	0.60	0.01	20.1	217	1.84
Zarzis	95862	0.21	0.10	0.00	19.8	216	1.84
Kasserine	826000	0.37	0.06	0.00	17.5	318	34.32
Tataouine	380000	0.79	0.65	0.00	20.0	123	0.92

Sheepd: sheep density (number/surface of region), Goatd: goat density, Cattled: cattle density, Temp(C°): Annual average temperature, Rain(mm): Annual average rainfall, Echhuman: human echinococcosis based on annual surgical incidence (ASI, new cases for 100,000 inhabitants).

#### Construction of genetic distances and reticulograms

The six bands from PCR 1121/1122 were each coded as present or absent for each *E. granulosus* egg isolate (MVSP 3.1, 2002). The average of Jaccard similarities of egg isolate banding patterns were calculated within and between regions. These average similarities were transformed into distances (as 1- average Jaccard similarity). T-REX software was used for constructing reticulograms of the genetic distance matrix [46]. The ADDTREE is one of the most frequently used methods for inferring phylogenetic trees. It reconstructs a phylogenetic tree structure starting from a star tree that contains n leaves associated with the objects and n-1 edges. The star tree is repeatedly developed by adding new internal nodes to it until a binary tree comprising of 2n-2 nodes (including n leaves and n-2 internal nodes) and 2n-3 edges is obtained [47]. A classical Neighbour joining reticulogram (NJ) was also constructed. Furthermore, a Mantel test was applied to show if there was any significant correlation between the genetic and geographic distances between studied areas using GENETIX software [48].

## Results

### Contamination index

**Figure 2:**
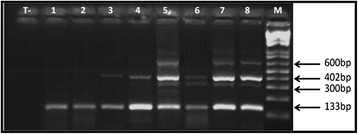
PCR banding patterns derived from *E. granulosus* eggs demonstrated the genetic polymorphisms of the EgG1HaeIII repeat. Lane T-: negative control, lanes 1,2: one band profiles (133 bp), lane 3,4: two bands profiles (133 bp, 402 bp), lanes 5, 7,8: four bands profiles (133 bp, 402 bp, 600 bp, 671 bp), lane 6: three bands profiles (133 bp, 402 bp, 600 bp), lane M: 100-bp DNA ladder marker (Promega).

**Figure 3:**
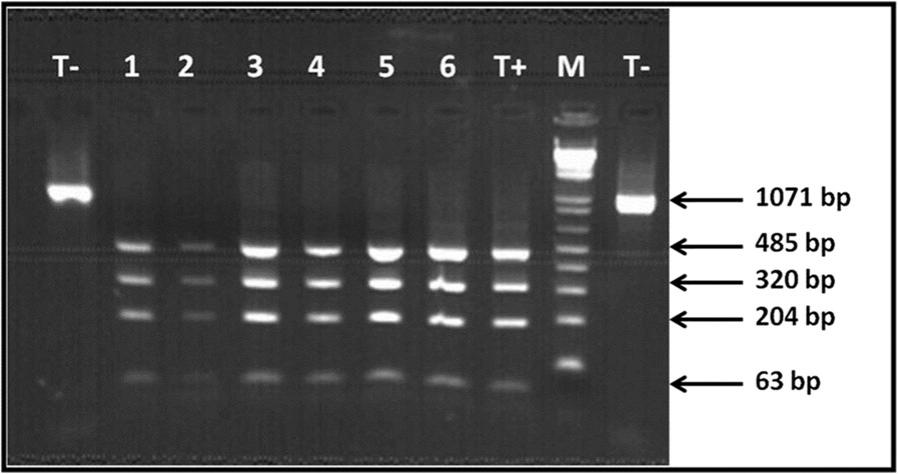
Restriction fragment length polymorphism analysis of nad1 gene amplified product. Lane T-: negative control (indigested nad1 PCR product), lanes 1–6: Identical patterns (485, 320, 204 and 63 pb fragments) for samples with one to six band profiles in 1121/1122 PCR, lane M: 1 Kb Plus DNA ladder marker (Invitrogen).

Among the 1095 faecal samples, 298 samples contained taeniid eggs. The ninety-three percent of taeniid samples (277 isolates) were identified as *E. granulosus* eggs. The banding patterns of the Eg1121/1122 PCR revealed profiles composed of one to six bands (133, 300, 402, 600, 671 and 940 bp) (Figure 2). The molecular analysis by PCR-RFLP of the nad1 gene produced identical patterns (four fragments of 485, 320, 204 and 63 bp) for all samples tested corresponding to *E. granulosus sensu stricto* profile (Figure 3). The overall contamination index of dog faeces by *E. granulosus* was 25.3% (277/1095) and different infection levels were observed between regions (p = 0.0006). The Metlaoui region had a significantly higher index than all other regions, followed by Djerba (Table 2). The other regions were similar.

**Table 2:** ***E. granulosus*** **contamination index of dog faeces in relation to regions**

**Region**	**Sousse n = 81**	**Monastir n = 95**	**Kasserine n = 132**	**Kef n = 36**	**Djerba n = 127**	**Zarsis n = 129**	**Metlaoui n = 392**	**Tataouine n = 103**
% of *E. granulosus* positive dog faeces	12.3^a^	9.5 ^a^	18.2 ^a^	8.3 ^a^	27.6^b^	17.8 ^a^	41.3^c^	14.6 ^a^

n = number of samples analyse.

The supercripts a, b, c indicate significant differences using Chi-square test, the level of significance was set at p< 0.05.

### Characteristics of the studied regions

**Figure 4:**
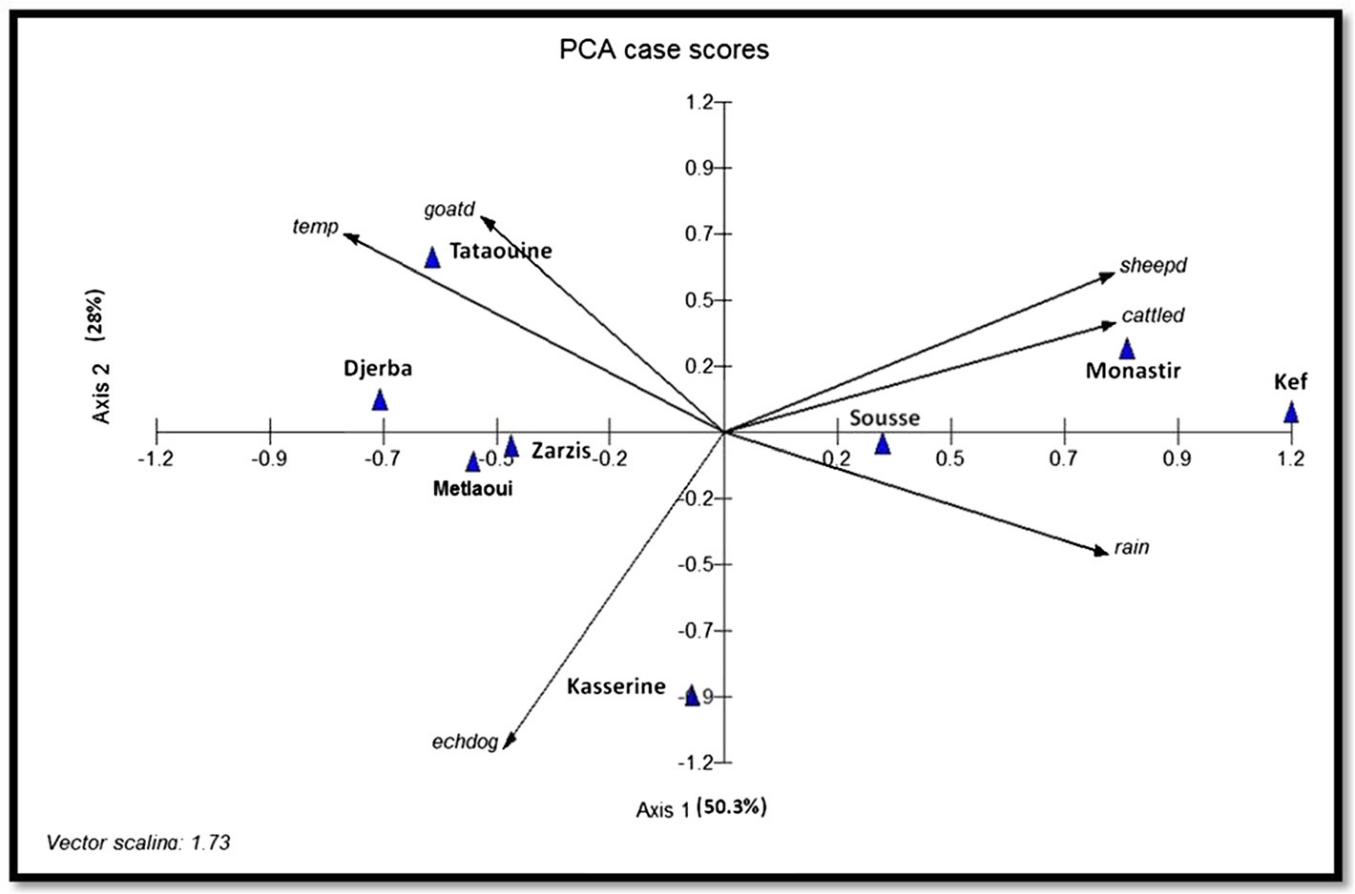
Dog echinococcosis and Tunisian region characteristics described by principal component analysis. echdog: dog faeces contamination index by *E. granulosus* eggs, temp: average yearly temperature, rain: average yearly rainfall, sheepd: sheep density (number/surface of region), cattled: cattle density, goatd: goat density.

**Figure 5:**
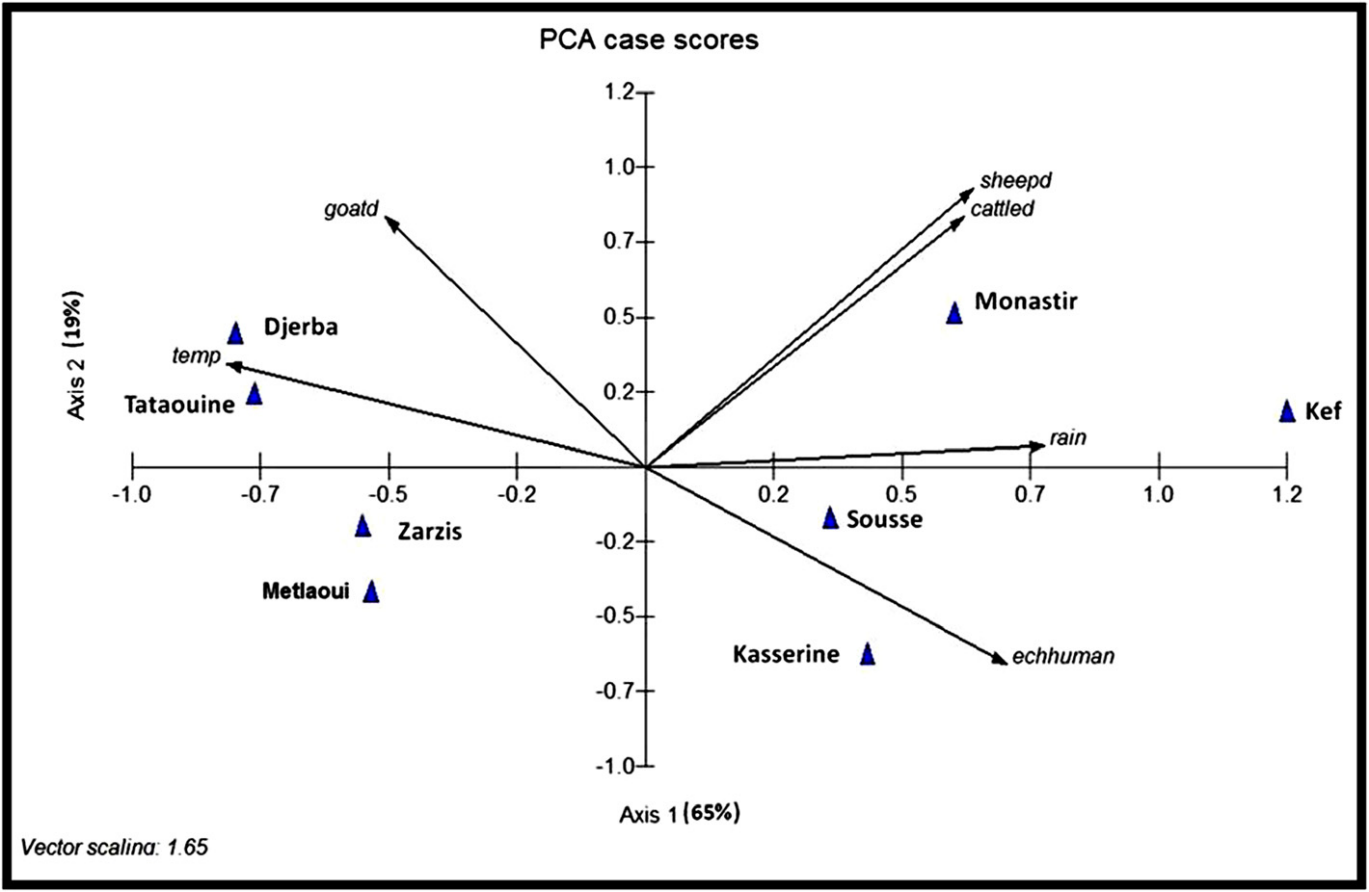
Human echinococcosis and Tunisian region characteristics described by principal component analysis. echhuman: human echinococcosis according to the annual surgical incidence, temp: average yearly temperature, rain: average yearly rainfall, sheepd: sheep density (number/surface of the region), cattled: cattle density, goatd: goat density.

The observed bioclimatic and geographic characteristics of the studied regions as well as sheep, goats and cattle densities and the prevalence of dog and human echinococcosis can be seen in Tables 1 and 2. The sheep density and to a lesser extent the cattle density, were negatively correlated to dog echinococcosis (p = 0.03) (Figure 4). The human echinococcosis incidence was significantly and negatively correlated to temperature (p = 0.001) and positively to rain (p = 0.03) (Figure 5). By including geographic and endemic/enzootic data in MVSP software, the PCA graphics showed the relationship of echinococcosis in dogs (echdog) and hydatidosis in humans (echhuman) (Figures 4 and 5) under natural conditions in the studied regions. CE in humans was more endemic in humid areas unlike canine echinococcosis which remained endemic even in arid regions. No relationship was observed between the contamination index of dogs and the livestock density within the regions studied.

### Genetic differentiation

**Figure 6:**
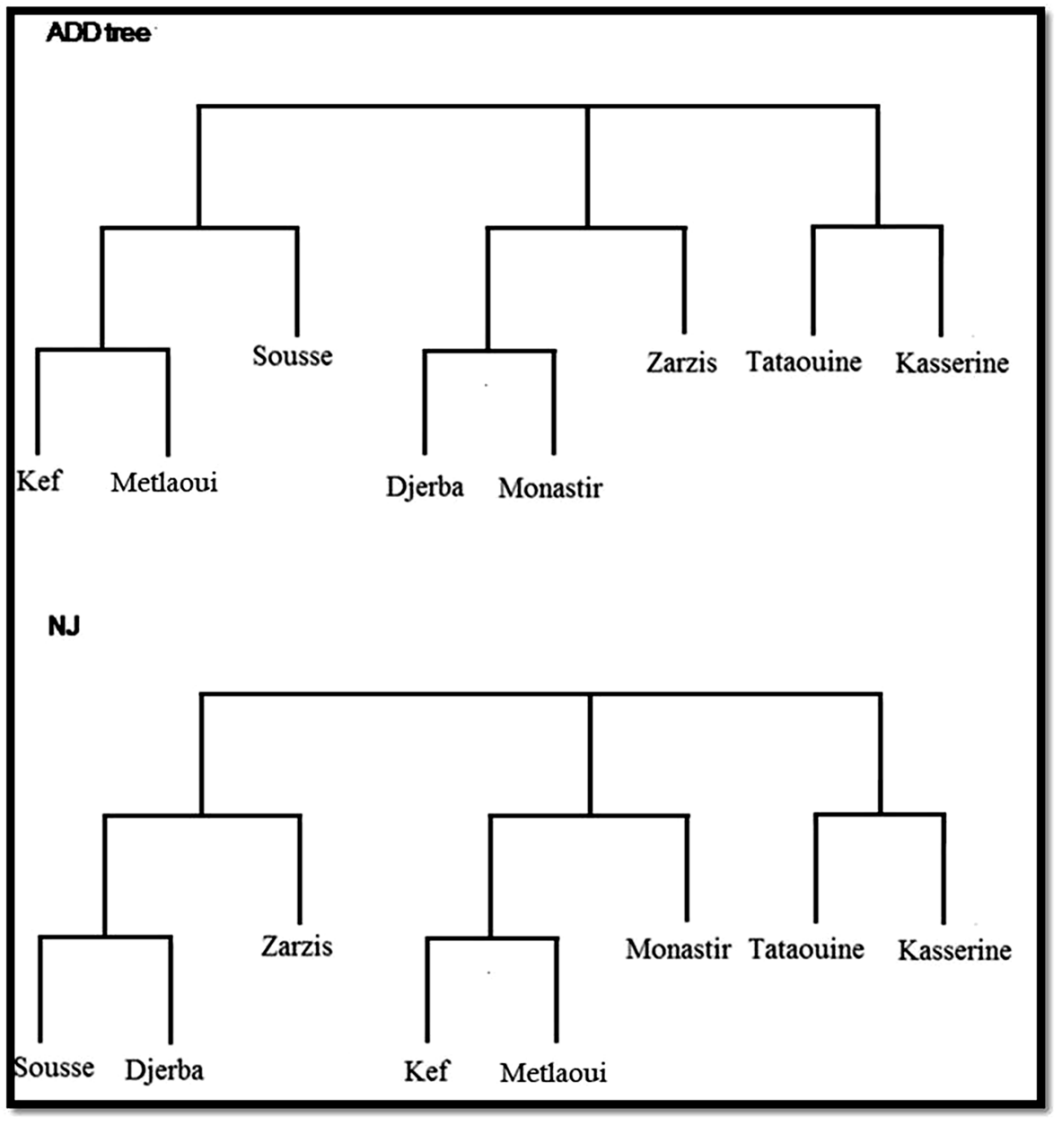
T-REX reticulograms with ADD tree and Neigbour Joining (NJ) methods based on genetic distances of the *E. granulosus* egg isolates of eight Tunisian regions.

**Figure 7:**
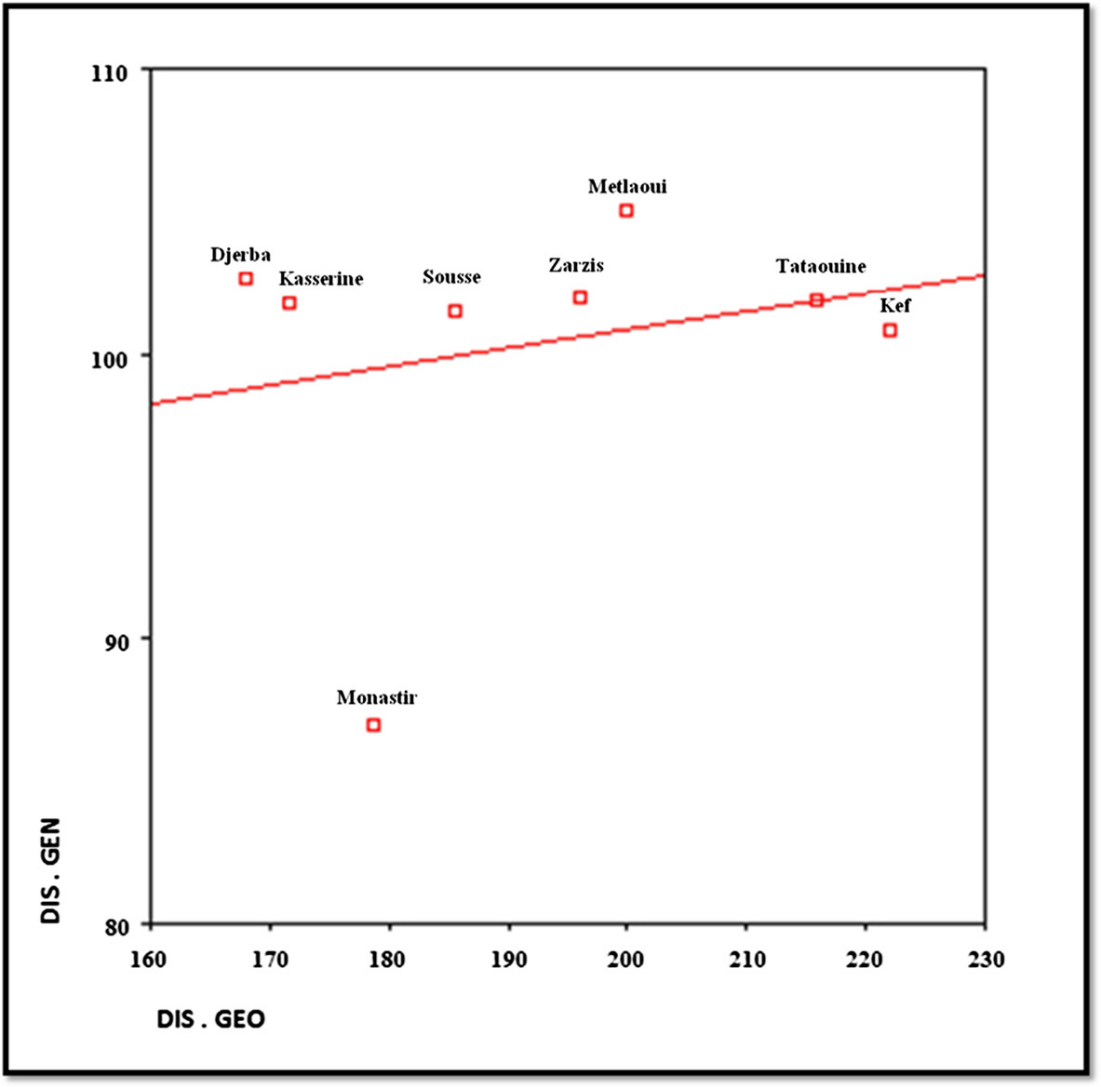
Genetic differentiation by distance (Mantel test). Average DIS. GEN for one site: Genetic distances, Average DIS. GEO for one site: Geographic distances in km.

The reticulograms by T-REX software using ADDTREE and Neighbour Joining methods on genetic distance matrix gave slightly different distributions (Figure 6). Two different stable groups were found in both reticulograms: Tataouine-Kasserine and Kef-Metlaoui. No significant correlation between the genetic distances based on PCR Eg1121/1122 results and the geographic distances was observed using the Mantel test (r = 0.06) (Figure 7).

## Discussion

The contamination index of dog faeces by *E. granulosus* eggs ranged from 8.3% to 41.3% depending on the region. The contamination index falls within the same range (prevalence: 9% to 41%) that was reported by Bentounsi *et al.* [49] in eastern Algeria based on necropsies in a similar environment. Similar values were also found in other neigbouring countries (Egypt, Lybia or Morocco) [8]. The PCA analyses based on human surgical incidence [6] and dog contamination index did not find the two to be related. Indeed, regions with high levels of canine echinococcosis have previously been found to be meso or hypoendemic for human echinococcosis (Metlaoui, Djerba and Zarsis). Moreover, hyper-endemic areas for hydatidosis, such as Kef, showed a low contamination index by *E. granulosus* eggs in dog faeces. Despite that dogs are involved in the parasite transmission, many other factors influence the human infestation, such as livestock management practices, hygiene levels, health education and finally lack of knowledge about the parasite life-cycle in the local population [19]. Home slaughtering is still practiced without any municipal veterinary supervision and it is probably an important source for dog infection. It should also be noted that the human incidence data used here was obtained few years before the present investigation. It is thus possible that the present human echinococcosis prevalence may have since changed [6]. The presence of *E. granulosus* eggs in the dog faeces was observed to spread without positive relation to livestock density of the studied regions (see Figure 4), showing a higher contamination index in isolates of warmer regions. The development of the dog parasite in dry areas with a limited number of herbivores might be related to the canine high densities in these regions (>1 per 5 inhabitants) rather than to climatic conditions since the survival of eggs is favored in humid areas [50]. The high contamination index of dog faeces observed in Djerba island (hydatidosis hypoendemic area) was unexpected. This may be due to the growing number of imported watchdogs into Djerba which defecated in the environment without faeces being collected. As reported by Kamiya et al. [51] in Japan islands, the moving of dogs from one place to another increases the risk of environmental contamination by *Echinococcus* eggs in uninfected or hypoendemic areas. In the present study, only the *E. granulosus* G1 genotype was found. Nevertheless, genetic polymorphisms were observed between these G1 isolates by the use of 1121/1122 PCR. The genetic distances were not related to geographic distances, which is indicative of local divergent evolutions rather than isolation by distance. This is in agreement with those reported by Oudni-M’rad et al. [52,53] who described significant genetic variation within the G1 genotype isolates of Sousse and Gafsa in Tunisia based on different genetic markers. No genetic structuration (genetic differences between regions) due to geographic distance could be found by the use of the Mantel test nor the TEX trees in NJ or the ADD tree applications. The G1 isolates could be alike in different regions since the intermediate hosts are transported from one farming region to another (slaughterhouse or livestock market).

## Conclusion

The relationship between human and dog infections is difficult to trace because a high environmental dog contamination index does not necessarily correspond to high prevalence in humans as transmission is strongly linked to human behavior and hygiene.
